# Salmagundi

**Published:** 1885-08

**Authors:** 


### SALMAGUNDI.

EDITED BY E. G. BETTY, D. D. S.

It is said that there are about 150 professors in the various German Universities whose ages range from 70 to 90 years.

"The queer statement is made that a police officer at Los Angols, Cal., has become delirious from the too frequent brushing of his teeth."

The Institute has awarded the biennial prize of $4,000 to Dr. Brown Sequard, for his discoveries in physiology.—*Cablegram, Paris, July 2nd.*

The fortieth annual commencement of the Ohio Dental College is out in a new dress and looks well. A good feature is an alphabetical list of the graduates corrected up to date.

"Gold dust has been found near Pioche, Nevada. Everybody in that section has lately been seeking for the spot where the sheep had been rolling." Glad to hear that a new supply of gold has been discovered, it may yet become cheaper than amalgam.

---

"A terrible retribution overtook a south Bend hen that imprudently swallowed a false tooth that dropped from the mouth of the old lady that was feeding it. Its head was cut off, and the false tooth had its revenge when it helped masticate the creature that had but a few hours before masticated it."

---

The new postal rates went into effect July 1st, and are as follows: Letters, two cents per ounce; merchandise, not sealed, one cent per ounce; and second-class matter mailed by others than publishers (newspapers, etc.,) one cent per each four ounces. We hope this will induce our friends to contribute odds and ends in goodly quantity, (quality always being standard) to Salmagundi, so that it will make things doubly interesting.

---

A germ emporium, so to speak, is to be opened on July 10 by the New York Polytechnic School of Medicine and Surgery. Bacilli of all kinds will be cultivated. A physician, who is taking an active interest in the project, says: "We will have facility for cultivating cholera bacilli, and shall make experiments to determine in what kind of soil these thrive best, in what temperature they live and at what temperature they die. Then they will be treated with all sorts of germicides, under various conditions, to show how they may be modified."—*Clipping.*

---

Mt. Sterling Ky., July 10, '85.

Editor "Sal."

I saw in the March number, something about a boy bustin, a gun, 'tis wrong.

July 4th. Boy, Gun, Fun,
Gun Bust, Dust.

I am glad that their is such a department in our literature, for there is a strong inclination in our profession to be long faced and serious, but if you knew our editor, "Sal.," as I do, and as good a "joak" on him as I could tell, it would cause you to smile audibly; but I dasent.

VAN.

---

L. W. MARSHALL M.D., of Nottingham, England, read a paper before the Midland Branch of the British Dental Association, on "Nutrition in Early Life as Affecting the Teeth." That is worthy of serious consideration, coming as it does from a medical man. It appears in the June number of the *Dental Record.* Among other things, he says: "Dental caries, I am thankful to see, has recently come before the Poor Law Authorities, at least in our district, and a new departure has been made by the appointment of a surgeon-dentist to the work-house school in that district. His duty will be to correct the hitherto routine practice of unlimited and early extraction. We medical men know full well how necessary this step is. Ill health, the result of imperfect assimilation, and consequent inability to do the work which might lift the individual from his humble position, is the inevitable termination to such neglect and error in practice. To you as dentists, this new field for observation cannot fail to give, as hospital work does to us, a larger area for enquiry."

---

ROSSBACH, of Jena, has been making some experiments, with a view to a better understanding of the movements of the stomach, derangements of which he believes to be at the bottom of many digestive troubles. The experiments were made upon dogs under the influence of profound morphia narcosis, and the results presented to the recent Congress fur Innere Medicine at Wiesbaden, and published in the Deutsche Med. Wochenschrift for April 30.

He has ascertained that as soon as the stomach is filled with food the peristaltic movements begin, at first feebly, increase gradually, and continue from four to eight hours. They occur only in the parts adjacent to the pylorus. The empty stomach is either entirely without motion, or exhibits only occasional or very feeble movements. The pylorus is closed during the entire period of digestion, and the emptying of the stomach begins suddenly when gastric digestion is, for the most part, accomplished. The duodenum is quite at rest during the entire period of gastric digestion.—*Medical News.*

